# The effects of feeding ferric citrate on ruminal bacteria, methanogenic archaea and methane production in growing beef steers

**DOI:** 10.1099/acmi.0.000180

**Published:** 2020-11-23

**Authors:** Brooke A. Clemmons, Liesel G. Schneider, Emily A. Melchior, Amanda K. Lindholm-Perry, Kristin E. Hales, James E. Wells, Harvey C. Freetly, Stephanie L. Hansen, Mary E. Drewnoski, Sarah J. Hartman, Phillip R. Myer

**Affiliations:** ^1^​ Department of Animal Science, University of Tennessee, Knoxville, Tennessee 37996, USA; ^2^​ USDA, ARS, U.S. Meat Animal Research Center, Clay Center, Nebraska, 68933, USA; ^3^​ Department of Animal Science, Iowa State University, Ames, Iowa 50011, USA; ^4^​ Department of Animal Science, University of Nebraska, Lincoln, Nebraska 68583, USA; ^†^​ Present address: Department of Animal and Range Science, New Mexico State, Las Cruces, NM 88003, USA; ^‡^​ Present address: Department of Animal and Food Sciences, Texas Tech University, Lubbock, TX 79409, USA

**Keywords:** beef, ferric citrate, methane, microbiome

## Abstract

Methane produced by cattle is one of the contributors of anthropogenic greenhouse gas. Methods to lessen methane emissions from cattle have been met with varying success; thus establishing consistent methods for decreasing methane production are imperative. Ferric iron may possibly act to decrease methane by acting as an alternative electron acceptor. The objective of this study was to assess the effect of ferric citrate on the rumen bacterial and archaeal communities and its impact on methane production. In this study, eight steers were used in a repeated Latin square design with 0, 250, 500 or 750 mg Fe/kg DM of ferric iron (as ferric citrate) in four different periods. Each period consisted of a 16 day adaptation period and 5 day sampling period. During each sampling period, methane production was measured, and rumen content was collected for bacterial and archaeal community analyses. Normally distributed data were analysed using a mixed model ANOVA using the GLIMMIX procedure of SAS, and non-normally distributed data were analysed in the same manner following ranking. Ferric citrate did not have any effect on bacterial community composition, methanogenic archaea nor methane production (*P*>0.05). Ferric citrate may not be a viable option to observe a ruminal response for decreases in enteric methane production.

## Introduction

Enteric methane (CH_4_) produced by cattle contributes to atmospheric greenhouse gas concentrations, and ruminant enteric fermentation is the second largest source of CH_4_ emissions from global livestock supply-chain emissions, accounting for 39 % of the total emissions [1]. Consequently, decreasing enteric methane produced by ruminants stands to lessen the impact of livestock-associated greenhouse gas emissions. Numerous studies have examined ruminant enteric and manure methane mitigation strategies and technologies, including the use of inhibitors [2], ionophores [3], plant bioactive compounds [4] and de-faunation [5], to name a few. However, results from these studies are variable and inconsistent, leading to difficulties in effective implementation of methane mitigation strategies. Thus, it is imperative to determine effective, consistent methods for decreasing methane emissions.

The use of alternative electron acceptors in diets may impact enteric methane, and the ferric citrate may act as this alternative electron acceptor when ingested by cattle. Ferric iron has routinely been studied because of its ability to decrease hydrogen sulfide gas (H_2_S) production in anaerobic systems by inhibiting sulphate reduction to sulfide [6, 7], and subsequently decreasing the amount of hydrogen available to sulfate-reducing bacteria (SRB). Ferric iron has a greater redox potential than sulphate and CO_2_, and can outcompete the electron acceptors of sulphate and CO_2_ for electrons [6]. However, as ferric iron is a relatively high-energy electron acceptor, its reduction is restricted by its solubility. Although most forms of ferric iron are relatively insoluble, chelation to citrate increases its solubility [8]. In the rumen, H_2_ gas produced during microbial fermentation is used as an energy source by methanogens, and thus methanogenesis may be inhibited by ferric citrate due to its thermodynamic favorability over CO_2_ for hydrogen. Indeed, recent wetland studies have demonstrated this, in which use of ferric citrate resulted in a decrease of methane production by 64 % in the field [9]. This method may, therefore, be an effective manner in which to decrease enteric methane production during microbial fermentation in beef cattle.

Although research in cattle has begun to examine use of ferric citrate as a methane mitigation strategy, few to date have determined the effects of feeding ferric citrate to beef cattle and its influence on the rumen microbiota, potentially impacting methane production [10, 11], and no studies have been conducted *in vivo* to evaluate the effect of ferric citrate on the ruminal microbial communities. Therefore, it was hypothesized that the addition of ferric citrate to the diet of growing beef steers would impact the ruminal environment via populations of bacteria and methanogenic archaea and thus decreasing methane production.

## Methods

### Animals and experimental design

All animals were sourced from the U.S. Meat Animal Research Center (USMARC) in Clay Center, NE, USA. Eight steers were housed in four partially covered concrete pens (two steers per pen) during a 16 day diet adaptation period. Cattle were then moved to a metabolism facility for the collection period where they were housed in individual stalls (87×214 cm^2^) equipped with automatic, individual water cups. Prior to the start of the study, steers were acclimated to close human contact and the metabolism facility for at least 6 weeks.

The experiment consisted of a replicated Latin square with four dietary treatments and four sample collection periods. Based on previous studies [10], and estimating the amount in the diet that would equate in terms of concentrations in the rumen fluid, dietary treatments consisted of 0, 250, 500 or 750 mg Fe/kg DM, with Fe being supplemented as ferric citrate and added to the diet in the ground corn carrier (Table 1). As a percentage of DM, diets consisted of 20 % dry-rolled corn, 5 % ground corn (as the carrier for the ferric citrate), 35 % chopped alfalfa hay, 30 % corn silage, 6 % dried distiller’s grains with solubles, and 4 % mineral and vitamin supplement with monensin (Steakmaker supplement; Table 1). Steers were stratified by body weight and assigned to a treatment. Each of the four periods was 21 days in length, consisting of 16 days of diet adaptation and 5 days of collections. Steers were weighed before the trial began and before each collection period when they were placed in individual metabolism stalls. Daily, each steer was offered 110 % of the previous day’s feed intake to ensure *ad libitum* intake was achieved. During this 5 day period, O_2_ consumption and CO_2_, and CH_4_ exchanges were measured for 24 h using a portable headbox respiration calorimeters. At the end of the 5 day collection period, steers were weighed before they were returned to their pen. At the end of each period when the steers were weighed, approximately 200 ml of rumen content was collected via an oesophageal tube and a vacuum pump. Immediately following sampling, rumen content was transferred to two 50 ml conical tubes and frozen. Samples were stored at −80 °C until processing.

**Table 1.** Diet with 0, 250, 500 or 750 mg Fe/kg DM, with Fe being supplemented as ferric citrate

Ingredient	% of dry matter
Dry-rolled corn*	20
Ground corn†	5
Chopped alfalfa hay	35
Corn silage	30
DDGS‡	6
Steakmaker supplement§	4
Salt	0.3

*Dry-rolled corn was decreased to 19.70 % of the diet in period 3 and 4 to account for the inclusion of NH_4_Cl.

†Ground corn used as the carrier for ferric citrate.

‡Dried distiller’s grains with solubles.

§Supplement contained (DM basis): 26.66 % crude protein; 3.39 % ether extract, 21.21 % neutral detergent fibre; 10.24 % acid detergent fibre; 15.46 % calcium; 0.356 % phosphorus; 0.500 % magnesium; 0.654 % potassium; 0.527 % zinc sulphate; 0.275 % sulphur; 0.002 % cobalt; 0.004 % iodine; 0.083 % selenium premix (0.2 % Se); 0.007 % vitamin A (1 000 000 IU g^−1^); 0.105 % vitamin E (500 IU g^−1^); monensin (746 grams/ton).

||Ammonium chloride added during period 3 and 4 (i.e. after day 42) to reduce phosphate crystals observed in urine.

### Measurement of gas production

Before each sampling and collection period, O_2_ consumption as well as CO_2_, and CH_4_ gases were measured by indirect calorimetry using eight portable respiration headboxes for 24 h using the procedure previously reported by Hales *et al*. [12]. At least three air turnovers were permitted before gas measurements were determined. The daily diet allotment of the animal was placed in each head box before gas collections were initiated, and the cattle typically consumed >85 % of the offered feed. Gas exchange was determined by pulling air through the headbox across a temperature-compensated dry test metre to determine airflow exiting the headbox. Air temperature and humidity were determined in real time. In order to form a composite air sample for the collection period for each individual headbox, proportional samples of background air entering the box and air exhausted from the headbox were collected in polyethylene-aluminum-Mylar laminate gas bags. Gas samples were analysed for O_2_, CO_2_ and CH_4_ according to Nienaber and Maddy [13], and specifically, CH_4_ was analysed using an infrared gas analysis system (AR-60A, Anarad, Santa Barbara, CA USA). Each headbox was calibrated for O_2_ consumed and CO_2_ produced by burning absolute ethanol with alcohol lamps before gas measurements were collected. The alcohol recoveries ranged from 98–101 % in all headboxes.

### DNA extraction and sequencing

Microbial DNA was extracted similarly to methods described by Yu and Morrison [14]. After the chemical and mechanical cell lysis, nucleic acids were precipitated using isopropanol. The DNA was purified with RNase and proteinase K treatment, followed by the use of QIAamp columns from the Qiagen DNA Stool Mini Kit (Qiagen, Hilden, Germany). Genomic DNA concentration was determined using a Nanodrop 1000 spectrophotometer (Thermo Scientific, Wilmington, DE, USA). Extractions were stored at −20 °C until sequencing library preparation. The DNA was amplified using PCR for 27 cycles as follows: 30 s denaturation at 95 °C, annealing for 1 min at 58 °C, and elongation for 90 s at 72 °C, with an initial denaturation for 5 min at 95 °C and a final elongation of 72 °C for 10 min. The V1-V3 hypervariable regions of the bacterial 16S rRNA gene was targeted for amplification. Modified universal primers 27F(5′-Adapter/ Index/ AGAGTTTGATCCTGGCTCAG) and 519R (5′-Adapter/ Index/ GTATTACCGCGGCTGCTG) including TruSeq indices and adapters were used with AccuPrime Taq high fidelity DNA Polymerase (Life Technologies, Carlsbad, CA, USA) to produce sequencing libraries [15]. Amplification products were quality checked with gel electrophoresis. Libraries were then purified using AmPure beads (Agencourt, Beverly, MA, USA), and quantified using a Nanodrop 1000 spectrophotometer (Thermo Scientific, Wilmington, DE, USA) and by real-time PCR on the LightCycler 480 system (Roche Diagnostics, Mannheim, Germany). The resulting libraries were sequenced using the 2×300, v3 600-cycle kit and the Illumina MiSeq sequencing platform (Illumina, San Diego, CA, USA) at the United States Meat Animal Research Centre (US MARC; Clay Centre, Nebraska, USA).

### Sequence-read processing and analyses

For bacterial community analysis, amplicon sequence reads were processed using the Quantitative Insights Into Microbial Ecology (QIIME) bioinformatics pipeline, version 1.9.1 [16]. Sequences were quality trimmed using the Galaxy server [17] and those with a score ≥Q30 were retained. Sequences that contained read lengths shorter than 300 bp were removed and adapters/index sequences were trimmed. Chimeric sequences were identified and filtered using usearch61 [18, 19]. To avoid biases generated by differences in sequencing depth, each sample was subsampled to an even depth of 60 000 sequences based on a sample with the lowest number of sequences after quality and chimeric filtering. Sequences classified as chloroplasts and mitochondria were removed. Sequences were clustered using UCLUST into species-level operational taxonomic units (OTU) against the Greengenes v13_8 16S rRNA database with a pairwise identity threshold of 97 % [16, 18]. In order for OTUs to be retained, they had to appear in ≥25 % of samples. Phylogenic trees were built with FastTree [20] to determine *α*- and *β*-diversity metrics. Then, *α*-diversity was analysed using observed species, Shannon diversity, PD whole tree, chao1, Simpson’s evenness E and equitability indices. Analysis of similarity (ANOSIM) was used to analyse *β*-diversity among treatments based on weighted and unweighted UniFrac distances and visualized using principal coordinates analysis (PCoA) in QIIME [21].

### Methanogen 16S rDNA

Following DNA extraction, real-time quantitative PCR (qPCR) was performed to determine the level of methanogen 16S rDNA. This was performed similarly to the established method by Freetly *et al*. [22]. Oligonucleotide primers used for qPCR analyses targeted eight methanogen groups: Methanomicrobiales (order), Methanobacteriales (order), *Methanosarcina* (genus), *Methanobacterium* (genus), *Methanobrevibacter ruminantium +Mbb. cuticularis*, and *Methanobrevibacter smithii +Mbb. wolinii +Mbb. thaueri +Mbb. gottschalkii +Mbb. woesei* as described in Freetly *et al*. [22]. Each DNA sample was amplified in triplicate. Quantitative PCR reactions included: 15 ng DNA template and 5 µl Sso Master mix (Bio-Rad), 10 µM each primer and adjusted to 10 ul with water. PCR was performed on a CFX384 thermal cycler (Bio-Rad, Hercules, CA, USA) under the following conditions: 3 min at 98 °C followed by 40 cycles of 98 °C for 15 s and 60 °C for 20 s. A melting curve analysis from 65–95 °C was performed after the amplification reactions were completed. To determine copy numbers for each sample, standard curves for each primer set were produced. Templates for the standard curve reactions were generated by cloning PCR amplicons produced using each primer set into the Topo vector and transformed into One Shot TOP10 competent cells (Topo TA cloning kit, Invitrogen, Carlsbad, CA, USA). The DNA from transformed *E. coli* cells was obtained by using the QIAprep spin miniprep kit (Qiagen, Hilden, Germany) and plasmid purity was confirmed by agarose gel electrophoresis. Concentrations of DNA were determined with a Nanodrop 1000 (Thermo Scientific, Wilmington, DE, USA) and gene copy numbers were calculated with the Thermo Scientific DNA copy number calculator tool (http://www.thermoscientificbio.com/webtools/copynumber/). Samples utilized as plasmid controls were diluted to 10^2^ through 10^8^ copies of plasmid copies μl^−1^. Calculations of copy numbers for each sample were performed using standard curve calculation in the Bio-Rad CFX Manager software (version 3.1; Bio-Rad, Hercules, CA, USA).

### Statistical analyses

Data were analysed for normality using the UNIVARIATE procedure in SAS 9.4, and normality was assumed using a Shapiro-Wilk statistic of ≥0.90 and visualization of histograms and plots of residuals. Normally distributed data were analysed using a mixed model ANOVA using the GLIMMIX procedure in SAS 9.4 (SAS Institute, Cary, NC, USA). Data that were not normally distributed were first ranked and then analysed using a mixed model ANOVA using the GLIMMIX procedure in SAS 9.4. The fixed effect of treatment was analysed and included random effects of period and animal. Significance was declared using *α*≤0.05.

## Results

### Bacterial community composition

**Fig. 1.**
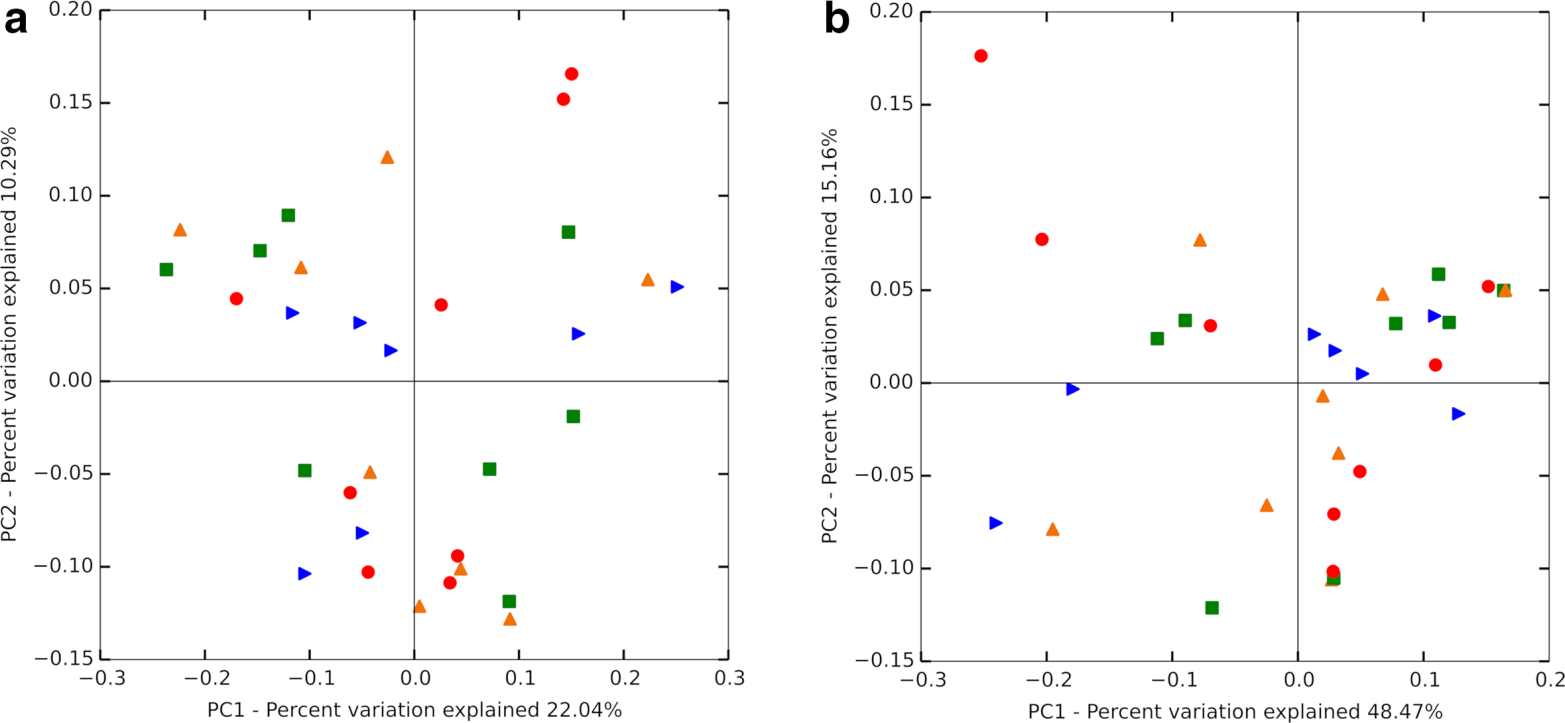
Principal coordinates analyses based on (a) unweighted and (b) weighted UniFrac distances using 9999 permutations. The level of ferric citrate inclusion is represented by differing symbols. Green square=0 mg, red circle=250 mg, blue arrow=500 mg and orange triangle=750 mg Fe/kg DM, with Fe being supplemented as ferric citrate.

**Fig. 2.**
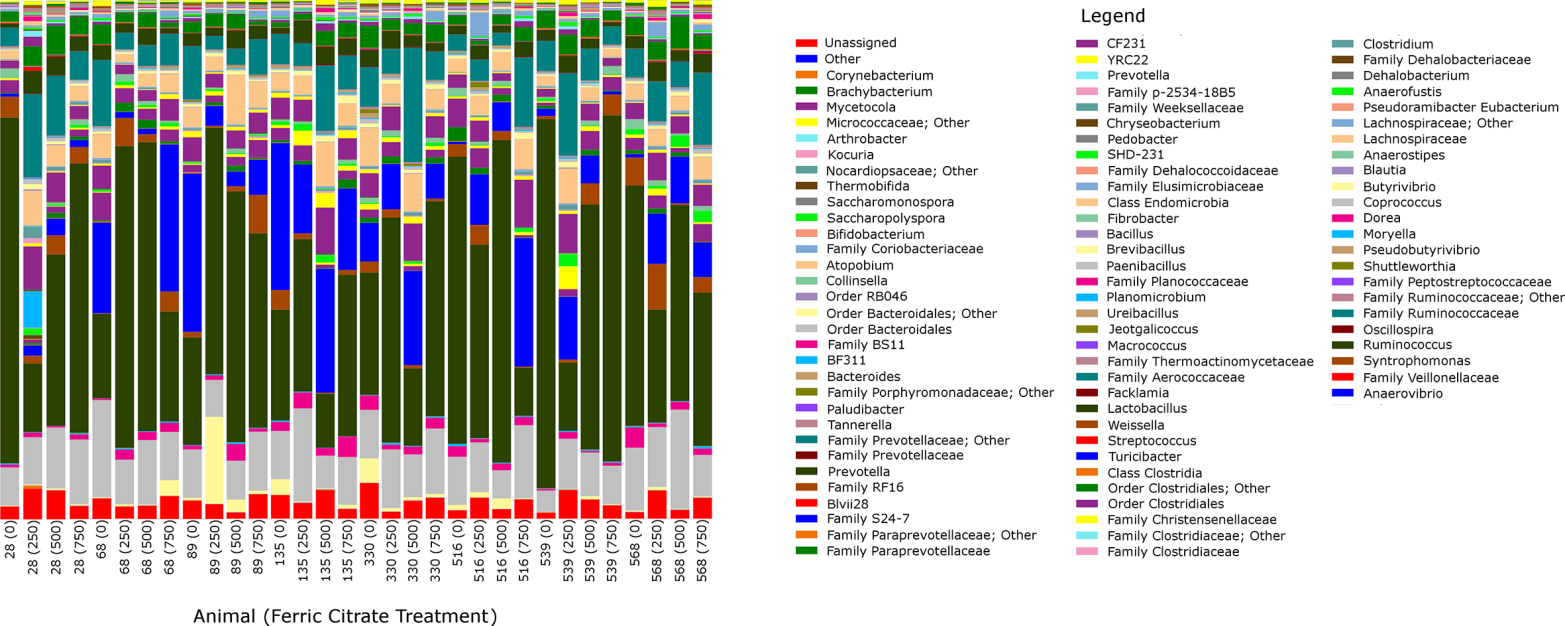
Bar chart of genera relative abundances (out of 100%). Animal IDs are listed with ferric citrate treatment amounts in parentheses (mg Fe/kg DM, with Fe being supplemented as ferric citrate).

Collectively, the sampled rumen contents of the eight steers resulted in a total of 4 211 015 overall reads after quality control and chimaera detection and removal. Individual samples generated an average of 131 594 cleaned sequence reads, ranging from 61 204 to 258 993 sequences. Within the total cleaned sequences, an average of 2822±39 OTUs were detected per sample. Good’s coverage indicated adequate coverage, with ≥0.98 coverage among all treatments (Table 2). The *α*-diversity metric Shannon’s diversity index was not different among treatments (*P*=0.31). Other *α*-diversity indices such as PD whole tree (*P*=0.33), chao1 (*P*=0.75), observed OTUs (*P*=0.26), Simpson’s evenness E (*P*=0.51) or equitability (*P*=0.33) were not different among treatments (Table 2). *β*-diversity did not differ among treatments based on weighted (R=−0.08, *P*=0.99) and unweighted (R=−0.09, *P*=0.98) UniFrac distances (Fig. 1). No bacterial genera were significantly different among treatments (*P*>0.05; Fig. 2).

**Table 2.** No significant differences in bacterial alpha diversity statistics by ferric citrate treatment*

Diversity metric	Ferric citrate treatment†				*P*-value
	0	250	500	750	
Good’s coverage	0.98±0.00	0.98±0.00	0.98±0.00	0.98±0.00	0.93
PD whole tree	87.59±2.30	89.96±2.04	90.08±1.84	91.24±2.45	0.33
Chao1	3615.70±89.00	3678.93±116.82	3665.25±115.25	3719.19±157.66	0.75
Observed OTUs	2737.75±85.80	2863.00±60.90	2813.00±53.70	2874.88±104.00	0.26
Shannon index	7.63±0.22	8.01±0.13	7.75±0.08	7.89±0.18	0.31
Simpson’s evenness E	0.02±0.00	0.02±0.00	0.02±0.00	0.02±0.00	0.51
Equitability	0.67±0.02	0.70±0.01	0.68±0.01	0.69±0.01	0.33

*P>0.05.

†Treatment in mg Fe/kg DM, with Fe being supplemented as ferric citrate.

### Archaeal abundance

Quantitative PCR was used to evaluate the copy number and type of the methanogen present in the rumen fluid from animals for each ferric citrate treatment and the control animals. The average copy numbers of methanogen order, genus and species for each treatment are presented in Table 3. No significant differences were observed among methanogenic archaeal quantities from ruminal fluid among treatments, including groups *Mbb. ruminantium +Mbb. cuticularis* (*P*=0.90), *Mbb. smithii +Mbb. wolinii + Mbb. thaueri + Mbb. gottshalkii + Mbb. woesei* (*P*=0.89), *Methanosarcina* (*P*=0.36), *Methanobacterium* (*P*=0.94), *Methanomicrobiales* (*P*=0.90), and *Methanobacteriales* (*P*=0.77).

**Table 3.** No significant differences observed in methanogenic Archaea by ferric citrate treatment*

Methanogenic Archaea†	Ferric citrate treatment‡				*P*-value
	0	250	500	750	
*Mbb. ruminantium + Mbb. cuticularis*	114 584±33 171	110 474±26 503	108 641±25 662	92 828±23 979	0.90
*Mbb. smithii + Mbb. wolinii + Mbb. thaueri + Mbb. gottshalkii + Mbb. woesei*	406 609±28 669	376 752±23 027	376 377±49 400	367 698±47 931	0.89
* Methanosarcina *	31 886 149±7 997 944	21 416 696±2 638 314	20 926 231±1 969 735	19 116 438±2 968 230	0.36§
* Methanobacterium *	179 484±19 795	164 754±11 524	170 557±21 664	169 744±21 833	0.94
Methanomicrobiales	306 826±31 788	284 164±22 768	283 451±30 049	290 379±28 939	0.90
Methanobacteriales	52 943 679±10 057 490	57 193 922±7 478 688	5 5068 829±12 485 778	44 890 414±8 512 105	0.77

*P>0.05.

†Copies per μl, determined by real-time quantitative PCR. Data represented as LSMeans.

‡Treatment in mg Fe/kg DM, with Fe being supplemented as ferric citrate.

§Based on ranked values.

### Methane production

**Fig. 3.**
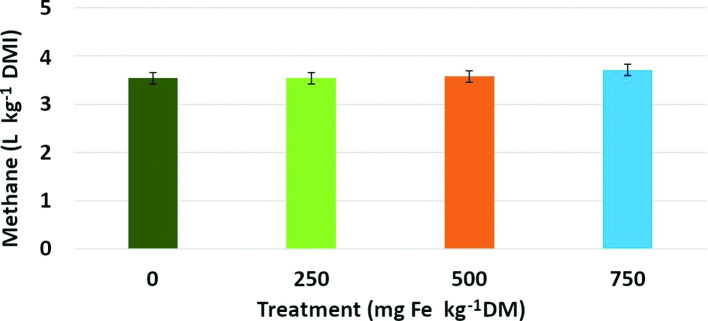
Methane emissions measured as litres per kg of dry matter intake in steers fed ferric citrate in the diet. Total emissions of methane did not differ between treatments (*P*>0.05). Error bars represent sem. Treatment in mg Fe/kg DM, with Fe being supplemented as ferric citrate.

Methane gas, measured as litres produced per kg of DMI per steer, was not significantly decreased by treatment with Fe being supplemented as ferric citrate at any level compared to the diet without ferric citrate (*P*>0.05, Fig. 3). Overall dry matter intake was not significantly altered across treatments (*P*>0.05).

## Discussion

In efforts to lessen anthropogenic methane emissions that arise from livestock production, alternative or novel methods continue to be developed and investigated to mitigate methane emissions in livestock. Cattle are the primary contributors to livestock-derived methane pollution, accounting for approximately 65 % of methane emissions from livestock [1, 23]. Decreasing methane not only may serve to lessen methane emissions, but may also improve cattle feed efficiency because methane production shunts carbons away from energy-production to produce methane [24]. Thus, identifying effective methods to decrease methane without negatively impacting rumen fermentative processes could result in both environmental and economic benefits for the cattle industry. This study examined ferric citrate as a potential methane-mitigation strategy by measuring methane production and ruminal archaeal and bacterial populations with increasing levels of ferric citrate.

In this study, ferric citrate did not have any significant effect on methane output. Ferric citrate has been used previously primarily *in vitro* to alter methane production. Wu and others conducted a study examining the effects of increasing levels of ferric citrate and ferric oxide (0, 25, 50, 100, 150 and 200 mg l^−1^ as Fe^3+^) on methane production, archaeal populations and sulphur-reducing bacterial (SRB) populations in rumen fluid culture from a Jersey cow on a total mixed ration [10]. In that study, neither ferric citrate nor ferric oxide significantly decreased methane production, though increasing amounts of ferric citrate tended to be associated with decreased methane production [10]. However, ferric citrate did alter archaeal and bacterial populations. At 25 mg l^−1^, ferric citrate decreased total bacteria. Further, every level of ferric citrate inclusion increased total archaeal populations, with greater archaeal populations occurring at greater levels of ferric citrate [10]. Given that methane production was not affected in the study by Wu and others, as well as the present study, these results may suggest that ferric citrate does not impact methane production. However, it is difficult to compare and contrast *in vitro* and *in vivo* studies as *in vivo* processes are not always accurately represented, which is evident by the lack of impact on the archaeal and bacterial populations in the current study.

One factor that may have affected the lack of significant results in this study may have been the amount of time animals underwent the treatment. In this study, the steers were on the diet and treatment for a total of 21 days; however, recent research suggests more time is warranted to observe differences in the ruminal microbial populations. A study conducted by Clemmons and others examined the ruminal bacterial populations of 50 steers for 10 weeks following a standard 2 week diet adaptation [25]. In that study, bacterial populations did not begin to stabilize to final populations until at least 4 weeks following the adaptation period, which was 6 weeks after the introduction to the new diet [25]. Although the present study was focused on the inclusion of ferric citrate as a treatment rather than a new diet, ruminal microbial populations may require more time than was provided in the present study in order for significant changes to be observed, both in microbial populations as well as methane production.

Monensin was also included in this study, which may also have affected the results. Monensin is an ionophore that acts to decrease methanogenesis by decreasing hydrogen-producing bacteria through disruption of ion exchange across the cell membrane [26, 27]. Because hydrogen is a substrate for methanogenesis, the decline in available hydrogen results in decreased methane production [26]. A study conducted by Thornton and Owens measured the effect of monensin on increasing levels of roughage (12 % acid detergent fibre [ADF; low], 27 % ADF [medium], and 40 % ADF [high]) with or without 200 mg of monensin [28]. Thornton and Owens found that monensin decreased methane production as ADF increased [28]. Because methanogenesis is primarily a result of microbial function, the monensin may have altered the microbial populations in the rumen decreasing methane production. Thus, the inclusion of monensin in the present study may have limited or masked any potential effects of ferric citrate that would have been observed otherwise. It should be noted, however, that monensin use is practical and common in the United States feedlot industry, and effects of ferric citrate on methane production in cattle not receiving an ionophore are not very relevant to the industry

In this study, ferric citrate did not significantly affect methanogenic archaea, bacterial community composition nor methane production. It is possible that the anticipated beneficial effects could not be observed when ferric citrate was used in conjunction with monensin. Additionally, the length of the treatment may not have been long enough to substantially alter the rumen microbiome and, in turn, methane production. Subsequent studies could assess use of ferric citrate without the use of monesin, for longer length of time, and in greater number of animals to possibly observe a ruminal repsonse to ferric citrate use for decreases in enteric methane production.
